# How do Malawian women rate the quality of maternal and newborn care? Experiences and perceptions of women in the central and southern regions

**DOI:** 10.1186/s12884-015-0560-x

**Published:** 2015-08-15

**Authors:** Christabel Kambala, Julia Lohmann, Jacob Mazalale, Stephan Brenner, Manuela De Allegri, Adamson S. Muula, Malabika Sarker

**Affiliations:** Institute of Public Health, Faculty of Medicine, University of Heidelberg, INF 324, Heidelberg, Germany; School of Public Health and Family Medicine, College of Medicine, University of Malawi, Blantyre, Private Bag 360, Chichiri, Blantyre 3, Malawi; Environmental Health Department, The Malawi Polytechnic, University of Malawi, Private Bag 303, Chichiri, Blantyre 3, Blantyre, Malawi; James P. Grant School of Public Health, BRAC University, 66 Mohakhali, Dhaka, 1212 Bangladesh

**Keywords:** Perceived quality of care, Maternal and newborn, Women’s health

## Abstract

**Background:**

While perceived quality of care is now widely recognized to influence health service utilization, limited research has been conducted to explore and measure perceived quality of care using quantitative tools. Our objective was to measure women’s perceived quality of maternal and newborn care using a composite scale and to identify individual and service delivery factors associated with such perceptions in Malawi.

**Methods:**

We conducted a cross-sectional survey in selected health facilities from March to May 2013. Exit interviews were conducted with 821 women convenience sampled at antenatal, delivery, and postnatal clinics using structured questionnaires. Experiences and the corresponding perceived quality of care were measured using a composite perception scale based on 27 items, clustered around three dimensions of care: interpersonal relations, conditions of the consultation and delivery rooms, and nursing care services. Statements reflecting the 27 items were read aloud and the women were asked to rate the quality of care received on a visual scale of 1 to 10 (10 being the highest score). For each dimension, an aggregate score was calculated using the un-weighted item means, representing three outcome variables. Descriptive statistics were used to display distribution of explanatory variables and one-way analysis of variance was used to analyse bivariate associations between the explanatory and the outcome variables.

**Results:**

A high perceived quality of care rating was observed on interpersonal relations, conditions of the examination rooms and nursing care services with an overall mean score of 9/10. Self-introduction by the health worker, explanation of examination procedures, consent seeking, encouragement to ask questions, confidentiality protection and being offered to have a guardian during delivery were associated with a high quality rating of interpersonal relations for antenatal and delivery care services. Being literate, never experienced a still birth and, first ANC visit were associated with a high quality rating of room conditions for antenatal care service.

**Conclusions:**

The study highlights some of the multiple factors associated with perceived quality of care. We conclude that proper interventions or practices and policies should consider these factors when making quality improvements.

**Electronic supplementary material:**

The online version of this article (doi:10.1186/s12884-015-0560-x) contains supplementary material, which is available to authorized users.

## Background

Good quality of care during pregnancy, childbirth, and the postnatal period is important for the health of mothers and their babies [1]. Predominantly preventive health care for pregnant women is typically provided through antenatal care (ANC) services in order to detect and treat potential health problems throughout the course of the pregnancy. ANC also offers the opportunity to develop a strong provider-client relationship and the exchange of important information that can result in improved obstetric outcomes [2, 3]. During labour and delivery, a woman requires constant monitoring and assistance from skilled birth attendants to successfully deliver the baby [1]. Postnatal care (PNC) services are critical to the health and survival of both the mother and her newborn, beginning immediately after birth until several weeks after. Poor PNC practices at this point in time may result in death or disability [4, 5].

Out of 30 million women who become pregnant in Africa every year, an estimated quarter of a million women die from pregnancy-related causes [6]. Nearly half of them die during delivery or the first week after giving birth, mainly because of complications such as bleeding, obstructed labour, eclampsia and hypertensive disorders [6]. At least 300,000 babies in Africa die each year during childbirth (as intrapartum stillbirths) from complications such as obstructed labour and another 290,000 babies born alive die from birth asphyxia complications [6]. The estimated maternal mortality ratio (MMR) and neonatal mortality rate (NMR) in Malawi is 675/100,000 births [7] and 33 deaths per 1,000 live births, respectively [8].

Although the majority of these deaths could have been prevented by skilled care during pregnancy, childbirth, and the immediate postnatal period, almost 60 percent of African women do not utilize the recommended maternal and newborn services, or give birth at home without skilled attendants [6]. While it has been acknowledged that women struggle to access the care they need because of family, community, and infrastructural barriers [9], women also often do not utilize maternal and newborn services due to the inadequate and poor quality of these services [2, 10, 11].

There is a growing consensus that the perceived quality of maternal and newborn services may be a key determinant of utilization of care and thus ultimately influence maternal and perinatal outcomes [12–15]. Women require high quality client-oriented care services that address their individual needs throughout pregnancy in order to ensure optimal health for them and their infants [16]. As such, calls are made for improvements in maternal and newborn health care quality and with a stronger focus on women-centred health care delivery [17]. Clients’ satisfaction with health care service quality together with their personal experiences and expectations in seeking health care seem to influence their perceptions, and thus are critical to both the success of the provider-patient interaction, as well as to reforming the health system [17–19].

Some studies have suggested that provider-patient interactions, i.e. general staff attitudes (e.g. friendliness, politeness, humility, respect, sympathy, non-discrimination, attention, trust, commitment to work, assurance of confidentiality and communication) influence clients’ perceptions [20–25]. Others have found that the hospital environment, i.e. room hygiene, comfort, and the availability of supplies and drugs influences clients’ perceptions [23, 25]. Furthermore, staff competency, hospital procedures, waiting time [20, 26–29], efficiency of the health workers [20, 28], effectiveness of health care [30, 31], consistency with local beliefs [22], personal privacy [21, 23], and the opportunity for a woman’s social/family support [32] have been suggested to influence clients’ perceptions.

While perceived quality of care is now widely recognized in health care as influencing service utilization, to our knowledge, there has been little research done to quantitatively explore clients’ perceived quality of maternal and newborn care [13, 16, 21, 24, 26, 27, 32]. Of the existing studies, some have failed to clearly distinguish the link between perceived quality of medical care and patient satisfaction [16, 26, 27]. Although the concepts differ, often times they have been used interchangeably or assessed to take place concurrently, making it unclear as to how perceived quality of care is measured [13, 17]. Furthermore, to our knowledge, no quantitative studies have been conducted on clients’ perceived quality of maternal and newborn care in Malawi.

This study intended to fill this existing gap in knowledge by measuring women’s perceived quality of maternal and newborn care services using a composite quantitative scale which addresses multiple facets of health service quality. In addition, the study looked into which individual and service delivery factors influence such perceptions.

## Methods

### Study design and setting

A cross-sectional survey was conducted in 33 health facilities of four districts in rural Malawi: Balaka in the southern region, and Ntcheu, Dedza and Mchinji in the central region. Balaka has a total population of 338,430, Dedza has 655,979, Ntcheu 499,936 and Mchinji has 494,011. The four districts together account for 13.26 % of the total population of Malawi, currently reported to be 15 million [33]. The health facilities were selected because they are the ones officially identified by the Ministry of Health as providers of emergency obstetric care (EmOC) services in the four districts. The study was conducted over a period of 3 months from March to May 2013.

### Sampling and data collection

A sample of 830 participants was successfully recruited for the survey. Convenience sampling was used to enroll women exiting maternal care services (ANC clinics, labor and delivery wards, PNC clinics). Women exiting the facility for other reasons than maternal care were excluded. In order to obtain sufficient analytical power, we aimed at interviewing at least 8 women for each service cohort (ANC, delivery and PNC) at each of the study facilities to retrieve a total minimum cohort size of 264. Each exiting woman was asked by the enumerator about their willingness to take part in the study. Interested participants were included into the study after informed consent. Trained enumerators spent a total of three days at each facility to conduct the interviews. Each interview lasted about 45 minutes and was conducted in the local language, Chichewa, using structured questionnaires administered with support from electronic data entry devices.

### Study tool

The structured questionnaire included only closed-ended questions and was divided into five sections which collected information on the participants’ socio-economic and demographic characteristics, their past and present pregnancy history, health service utilization, their personal experiences with receiving maternal care services at the facility, and their perceptions of quality of care.

Perception of health care quality was assessed using a psychometric scale which was developed in a theoretically driven way. Wilde, Starrin, Larsson and Larsson’s theoretical model postulates that clients’ perceptions of what constitutes good quality care are basically formed by the resources available to the health service organisation and the patient preferences. These two main factors further influence the extent to which socio-cultural norms, expectations, and encounters with the service structures, and experiences in receiving care are individually perceived [19]. Individual perceptions of care quality are therefore based on what a client considers important to his or her clinical management [25], as well as the interpersonal relations during the provider-patient encounter [20–22, 25, 26], the structural and administrative conditions at the facility [22, 25], and the medical and social competence of the care givers [20–23, 26–32]. Based on this theoretical model [19] and building upon prior empirical research evidence [20–22, 25–29, 32, 34], we constructed a scale to measure three dimensions of health care perception: the interpersonal relations (i.e. clients’ experience with the socio-cultural atmosphere during the provider-client interaction), the conditions of the examination rooms (i.e. clients’ experience with the physical-technical conditions of the health service environment), and the nursing care services (i.e. clients’ experience with the medical-technical competence of the caregivers).

Each perception dimension was measured with a psychometric, Likert-type scale, encompassing a series of short statements (e.g. ‘The health worker listened to me’, ‘She/he behaved in a gentle manner’, ‘I was set up comfortably’, ‘The room was clean and hygienic’) that were adjusted to each of the three service cohorts: 27 items for women exiting each ANC or PNC clinics, and 34 items for women exiting the delivery wards. As part of the overall questionnaire, women were asked to rate each statement based on their experience with their respective health service visit at the day of interview. Each statement was read to the woman by the research enumerator. Women then indicated their level of agreement using a visual 10-point scale (10 indicating complete agreement and 1 indicating complete disagreement).

**Fig. 1:**
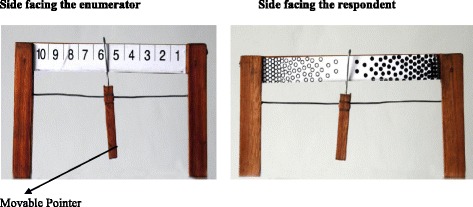
Schutte Scale for rating the perceived quality of care

To ease data collection among a population of primarily illiterate women, we used a hand-held rating scaling instrument which has tabulation from 1 to 10 on one side and circular patterns on the other (see Fig. 1). The densely populated dark coloured circles correspond to 10 on the tabulated side, and the densely populated light coloured circles corresponded to 1. During the interview, the participant held the scale in a way that the tabulated side faced the enumerator while the patterned side faced the participant. After each statement was read out by the enumerator, the participant would respond by matching the location of the pointer to her level of agreement or disagreement with the statement. The enumerator would then record the corresponding numerical value. The measurement instrument and the technique were adapted from De Wet Schutte who developed it for use in the assessment of priorities when identifying community needs in development projects [35].

The statements for measurement of each of the three dimensions of perception were adapted from a quality of care perception scale previously used in Burkina Faso to assess women’s perceptions of delivery services [34]. We first translated these statements from French into English and then into the local language, Chichewa. We then adapted the scale to the local context of Malawi, changing and refining a number of statements, and we adjusted it to be able to measure perceived quality of care also for ANC and PNC services. This process led us to develop three similar, yet distinct scales.

Each scale was designed to measure three dimensions of perceived service quality on:

1. Interpersonal relations between the health worker and the woman during the clinical encounter, made up of 10 statements for ANC and PNC, and 15 statements for delivery;
2. Conditions of the examination room, made up of 7 statements for ANC and PNC and 8 statements for delivery;
3. Nursing care services, made up of 10 statements for ANC and PNC and 11 statements for delivery.

### Analytical approach

Three statistical approaches were used in the analysis. First, a pre-analysis was conducted to test the validity and reliability of the newly adapted psychometric scale to measure perceived service quality. Specifically, for each perceived quality of care scale (ANC, delivery, PNC), Confirmatory Factor Analysis (CFA) was conducted to confirm the structure of the psychometric scale, i.e. whether the data supports the intended assignment of the scale statements to the quality of care dimension. In addition to this investigation of the factor analytic structure of the overall scales, Cronbach’s alpha (α) was calculated on each perceived quality dimension subscale for each service to determine the reliability of rated statements retained in each perception scale. The CFA results as well as α statistics are presented in the result section.

Second, descriptive univariate analyses were performed to inspect frequency distribution of variables that we selected as explanatory variables (Table 1 shows the specific variables). While most of the variables are self-explanatory, we measured woman’s wealth using household asset ownership i.e. sewing machine, television, radio, and bicycle; characteristics of dwelling house i.e. type of materials used for the wall, roof and floor, source of electricity, source of drinking water, type of toilet facility; ownership of agricultural assets i.e. farm land, goats, sheep, pigs and poultry. Principal Component Analysis (PCA) was used to generate a household wealth index from these variables based on which participants were assigned to wealth quartiles, with the categories labelled from 1 to 4 (1 being the poorest and 4 the wealthiest) [36].

**Table 1:** Explanatory variables and their distribution in the sample

Variable	ANC (N = 388)		Delivery (N = 230)		PNC (N = 230)	
	N	%	N	%	N	%
Woman's age						
<20	91	23.45	41	20.2	47	20.43
20-29	205	52.84	118	58.13	132	57.39
≥30	92	23.71	44	21.67	51	22.17
Woman's marital status						
Unmarried	9	2.32	14	6.9	13	5.65
Married	379	97.68	189	93.1	217	94.35
Woman's religion						
Non-Christian	35	14.18	25	12.32	48	20.87
Christian	335	85.82	178	87.68	182	79.13
Woman's literacy						
Illiterate	131	33.76	69	33.99	89	38.7
Literate	257	66.24	134	66.01	141	61.3
Woman's wealth						
1-Poorest	98	25.26	51	25.12	58	25.22
2	97	25	54	26.6	57	24.78
3	96	24.74	48	23.65	58	25.22
4-Least poor	97	25	50	24.63	57	24.78
Parity						
1	121	31.19	66	32.51	76	33.04
2-3	135	34.79	74	36.45	86	37.39
>3	132	34.02	63	31.03	68	29.57
Number of children						
No child	137	35.31	0	0	0	0
1-3 children	189	48.71	149	73.4	178	77.39
>3 children	62	15.98	54	26.6	52	22.61
History of miscarriage						
No miscarriage	333	85.82	180	88.67	198	86.09
Had miscarriage	55	14.18	23	11.33	32	13.91
History of still birth						
No stillbirth	371	95.62	197	97.04	221	96.09
Had still birth	17	4.38	6	2.96	9	3.91
History of Premature birth						
No premature birth	365	94.07	191	94.09	215	93.48
Had premature birth	23	5.93	12	5.91	15	6.52
Number of ANC visits						
First visit	181	46.65	NA	NA	NA	NA
>1 visit	207	53.35	NA	NA	NA	NA
Method of delivery						
Vaginal	NA	NA	190	94.53	NA	NA
C-section	NA	NA	7	3.48	NA	NA
Vacuum/forceps	NA	NA	4	1.99	NA	NA
Number of ANC visits at delivery						
0-3 ANC visits	NA	NA	122	60.4	NA	NA
>3 ANC visits	NA	NA	80	39.6	NA	NA
Length of stay before delivery						
0-2 days	NA	NA	170	83.74	NA	NA
≥2 days	NA	NA	33	16.26	NA	NA
Length of stay after delivery						
0-1 day	NA	NA	120	59.11	NA	NA
≥1 day	NA	NA	83	40.89	NA	NA
Mode of transportation						
Walked	232	59.79	66	32.51	154	66.96
Motorized transportation	156	40.21	137	67.49	76	33.04
Wait time						
≤1 hour	298	76.8	181	89.16	179	77.83
>1 hour	90	23.2	22	10.84	51	22.17
Self introduction by HW						
Not done	231	59.54	142	69.95	129	56.09
Done	157	40.46	61	30.05	101	43.91
Explanation of exam procedures (ANC, n = 380; Delivery, n = 121; PNC, n = 168)						
Not explained	95	25.00	13	10.74	55	32.74
Explained	285	75.00	108	89.26	113	67.26
Explanation of medicine purpose & how to take (ANC, n = 366; Delivery, n = 167; PNC, n = 108)						
Not explained	63	17.21	61	36.53	22	20.37
Explained	303	82.79	106	63.47	86	79.63
Explanation of blood specimen purpose (ANC, n = 267; Delivery, n = 85; PNC, n = 53)						
Not explained	20	7.49	23	27.06	6	11.32
Explained	247	92.51	62	72.94	47	88.68
Consent seeking (ANC, n = 384; Delivery, n = 201; PNC, n = 183)						
Not sought	104	27.08	82	40.8	63	34.43
Sought	280	72.92	119	59.2	120	65.57
Encouragement to ask questions						
Not encouraged	143	36.86	123	60.59	100	43.48
Encouraged	245	63.14	80	39.41	130	56.52
Encouraged to have a guardian						
Not encouraged	180	46.39	101	49.75	107	46.52
Encouraged	208	53.61	102	50.25	123	53.48
Confidentiality						
Not kept	24	6.19	17	8.37	30	13.04
Kept	364	93.81	186	91.63	200	86.96
Bp measurement ANC & PNC						
Not taken	147	37.89	NA	NA	133	57.83
Taken	241	62.11	NA	NA	97	42.17
Bp measurement before delivery						
Not taken	NA	NA	76	37.44	NA	NA
Taken	NA	NA	127	62.56	NA	NA
Bp measurement after delivery						
Not taken	NA	NA	116	57.15	NA	NA
Taken	NA	NA	87	42.86	NA	NA
Baby weight measurement						
Not taken	NA	NA	NA	NA	52	22.61
Taken	NA	NA	NA	NA	178	77.39

NA in all field of a cell means that this variable does not apply for the respective service

Third, bivariate analyses were conducted for each of the three service cohorts (ANC, delivery and PNC). For each service and perceived quality dimension subscale, a score was calculated as the un-weighted mean of a woman’s responses to the statements pertaining to the respective service and perceived quality dimension subscale to create the main outcome variables. Bivariate associations between each of the explanatory variables in Table 1 and each of the perception score (main outcome variables) were analysed using one-way analysis of variance (ANOVA) [37].

It should be noted that aspects of the service delivery process were also used as explanatory variables (Table 1). These variables by themselves are indicators commonly used in the assessment of quality of care (e.g. provider’s self introduction, provider explaining clinical procedures to client, provider seeking consent prior to medical intervention, provider ensuring client confidentiality). As these variables are based on a patient recalling the observation of certain processes they have not been included in the computation of the perception scores. Only information based on patient experiences as retrieved by the individual rating of validated statements were included in the scores used as outcome variable (see Additional files 1, 2 and 3).

Since there is an assumed clustering given that the study participants were sampled from specific health facilities (i.e. 33 clusters), we checked for clustering effect by use of an Intraclass Correlation (ICC) [38–40]. However, we found that the rho was small, ranging from values of 0.03 to 0.09 across all services (ANC, delivery, and PNC) and all dimensions of perceived quality (interpersonal relationships, service environment, and nursing care services). The one exception was a rho of 0.2 for the care giver competency dimension on PNC. As such, we ignored clustering effects in our analysis [38]. Stata IC version 13 (StataCorp LP, Texas) was used to analyze the data.

### Ethical consideration

Approval to conduct the survey was obtained from the Ethical Commission of the Medical Faculty at Heidelberg University (protocol number S-256/2012) and from the Malawi College of Medicine Research and Ethics Review Committee (protocol number P.02/13/1338). Before collecting data in the health facilities, permission was sought from the District Health Officers and from the relevant health facility authorities. Informed consent was obtained from all participants. A consent form written in the local language, Chichewa, was required to be read, understood, and signed before an interview commenced. For those participants that did not know how to read and/or write, interviewers read out the informed consent statement, and finger prints were accepted as signatures. All interviews were conducted on the facilities’ premises, but in a secluded, pre-arranged place to ensure privacy and confidentiality.

Since data was collected through electronic devices, data from each device was uploaded to a secure web server which was later downloaded to be stored securely on a local server. Thereafter, all data were deleted from web-accessible location and the electronic devices.

## Results

The survey was administered to a total of 821 women (388 in the ANC cohort, 203 in the delivery cohort and, 230 in the PNC cohort). The total number of participants from each district was as follows: Balaka (173), Dedza (240), Mchinji (180), and Ntcheu (229). Seven women declined to participate and two women discontinued the interview. The mean age (in years) for ANC was 25.45 (SD = 9.58), 25.70 (SD = 15.57) for delivery, and 27.13 (SD = 14.00) for PNC. Further details on the participants’ characteristics are presented in Table 1.

Service utilization: Of the 388 women interviewed after their ANC consultations, only 40 (19 %) had started their ANC visits in the first trimester. For the 203 women interviewed when exiting delivery wards, 190 (95 %) reported to have delivered normally, and no one reported to have experienced a neonatal death. Most women 120 (59 %) reported to have stayed at the facility for only 0–1 day after delivery. Over half of all women interviewed after delivery (60 %) had not attended the recommended minimum of 4 ANC clinics during their pregnancy. Of the 230 participants in the PNC exit interview, 137 (60 %) went for their first postnatal check within 7 days after giving birth. One hundred ninety six (85 %) of these reported to have delivered at the facility and only 34 (15 %) reported to have delivered elsewhere.

Participants’ experiences at the facility: Generally, women do not wait longer than an hour before they are attended to by the health worker. Only 23 %, 11 % and 22 % of the participants reported to have waited for one hour or longer before receiving ANC, PNC and delivery services respectively. While most participants were attended to quickly, many reported that the health workers did not introduce themselves during the clinical encounter (60 % for ANC; 70 % for delivery; and 56 % for PNC). Of the 388 women interviewed after their ANC consultations, 8 (2 %) were not examined, 22 (6 %) did not receive medication, 121 (31 %) did not get a blood test, and 4 (1 %) did not receive any other procedures that required a consent. Similarly, of the 203 women interviewed after delivery, 82 (40 %) were not examined, 36 (18 %) did not receive medication, 118 (58 %) did not get a blood test, and 2 (1 %) did not receive any other procedures that required a consent. Of the 230 women interviewed after their PNC consultations, 62 (27 %) were not examined, 122 (53 %) did not receive medication, 177 (77 %) did not get a blood test, and 47 (20 %) did not receive any other procedures that required a consent (Table 1 provides further details on the participants’ experiences at the facility).

Reliability of the Quality of Care Perceptions scales: CFA on each of the three scales gave a statistically significant chi-square result at the probability level of <0.001. These results would have led us to reject our hypothesis that this model is a good representation of actual client perception. However, the resulting goodness of fit statistics indicated adequate fit of the data to the structural assumptions of the scale for all three services. Specifically, we found Root Mean Square Error of Approximation (RMSEA) [41] of <0.05 as well as Comparative Fit Index (CFI) and coefficients of determination (CD) of 0.80 and 0.98 respectively. Cronbach’s α’s calculated on each perceived quality dimension subscale for each service confirm the CFA findings: α for the interpersonal relations subscales was found to be 0.83 for the ANC cohort, 0.87 for the PNC cohort and 0.85 for the delivery cohort. For the conditions of the examination and delivery room subscale, α was found to be 0.73 for the ANC cohort, 0.82 for the PNC cohort and 0.80 for the delivery cohort. For the general quality of nursing care services subscale, α was found to be 0.72 for the ANC cohort, 0.86 for the PNC cohort and 0.86 for the delivery cohort. α can be interpreted as the average inter-statement correlation. By convention, α’s of 0.7 and higher are acceptable and indicate that all statements do in fact measure one dimension, rather than several ones [42]. Results from the CFA and Cronbach’s α thus indicate that the intended grouping of the statements onto three dimensions (interpersonal relations, conditions of the examination rooms, general quality of nursing care services) is valid.

Participants’ perceptions of the quality of ANC, delivery and PNC care: The mean perception scores on interpersonal relations were 9.93 (SD = 1.7) among women leaving ANC clinics, 9.04 (SD = 1.8) among women leaving the labour and delivery service and 8.94 (SD = 1.8) among women leaving PNC clinics. The mean perception scores in relation to conditions of the examination rooms were 9.35 (SD = 1.4) among women leaving ANC clinics, 9.34 (SD = 1.5) among women leaving the labour and delivery service, and 9.15 (SD = 1.5) among women leaving PNC clinics. The mean perception scores on the general quality of nursing care services were 9.04 (SD = 1.5) among women leaving ANC clinics, 9.05 (SD = 1.2) among women leaving the labour and delivery, and 8.77 (SD = 1.8) among women leaving PNC clinics.

### Factors associated with perceived quality of interpersonal relations (provider-patient interaction)

Bivariate analysis results of the associations between the perceived quality of interpersonal relations and the explanatory variables are presented in Table 2. Literacy, mode of transportation used to travel to the facility, self-introduction by the health worker, explanation of examination procedure, explanation of medication, explanation of the purpose of blood specimen, consent seeking, encouragement to ask questions, assurance of confidentiality by the health worker and blood pressure (BP) measurement were all statistically significantly associated with perceived quality of ANC services (i.e. p < 0.05). Specifically, survey participants rated the quality of interpersonal relations higher when literate compared to illiterate, when having been transported to the facility by means of a motorized vehicle as opposed to walking, and when the provider had introduced themselves, explained procedures, purpose of medication and blood specimen, sought consent, encouraged to ask questions, ensured confidentiality, and measured blood pressure, as opposed to not having done so, respectively. Perceived quality of delivery services was statistically significantly associated with method of delivery, confidentiality being protected, and an offer to have a guardian by one’s side (i.e. p < 0.05). Specifically, women who had delivered via C-section rated the quality of the interpersonal relationship lower than women who had delivered vaginally or by vacuum/forceps. Women who had been encouraged to ask questions and to have a guardian by their side, as well as who were ensured of their confidentiality, rated the quality of the interpersonal relationship higher than women who had not experienced these treatments. Perceived quality of PNC services was found to be significantly associated with self-introduction by health workers, explanation of examination procedure, explanation of medication, explanation of the purpose of blood specimen, consent seeking and explanation of the purpose of the blood specimen (i.e. p < 0.05). Specifically, women rated the quality of the interpersonal relationship more highly if the health worker had done the above, rather than not.

**Table 2:** Bivariate associations between the perceived quality of interpersonal relations and the explanatory variables

Variable	ANC (N = 388)			Delivery (N = 203)			PNC (N = 230)		
	N	Mean	p-value	N	Mean	p-value	N	Mean	p-value
Age			0.128			0.609			0.970
<20	91	8.84		41	8.95		47	8.97	
20-29	205	9.07		118	9.02		132	8.94	
≥30	92	9.14		44	9.17		51	8.91	
Marital status			0.951			0.425			0.906
Unmarried	9	9.01		14	9.26		13	8.98	
Married	379	9.03		189	9.03		217	8.94	
Religion			0.918			0.615			0.499
Non-Christian	55	9.05		25	8.94		48	9.04	
Christian	333	9.03		178	9.06		182	8.91	
Literacy			0.002			0.249			0.391
Illiterate	131	8.80		69	8.92		89	8.85	
Literate	257	9.15		134	9.10		141	8.99	
Wealth			0.118			0.379			0.662
1 (poorest)	98	8.91		51	8.93		58	8.77	
2	97	8.99		54	9.17		57	8.99	
3	96	9.26		48	9.17		58	8.95	
4 (wealthiest)	97	8.98		50	8.89		57	9.04	
Parity			0.471			0.967			0.879
1	121	8.97		66	9.07		76	8.97	
2 to 3	135	9.00		74	9.02		86	8.96	
>3	132	9.13		63	9.04		68	8.88	
Number of children			0.228			0.859			0.526
No child	137	8.91		NA	NA				
1-3 children	189	9.09		149	9.03		178	8.97	
>3 children	62	9.14		54	9.06		52	8.84	
Miscarriage			0.229			0.460			0.378
No miscarriage	333	9.06		180	9.02		198	8.97	
Had miscarriage	55	8.87		23	9.20		32	8.76	
Still birth			0.560			0.368			0.857
No stillbirth	371	9.04		197	9.05		221	8.94	
Had stillbirth	17	8.88		6	8.66		9	8.87	
Premature birth			0.947			0.920			0.164
No premature birth	365	9.03		191	9.04		215	8.91	
Had premature birth	23	9.05		12	9.07		15	9.36	
Number of current ANC visit			0.048			NA			NA
First visit	181	9.15		NA	NA		NA	NA	
>1 visit	207	8.93		NA	NA		NA	NA	
Method of delivery			NA			0.046			NA
Vaginal	NA	NA		190	9.07		NA	NA	
C-section	NA	NA		7	8.06		NA	NA	
Vacuum/forceps	NA	NA		4	9.10		NA	NA	
Number of ANC visits, delivery			NA			0.084			NA
0-3 ANC visits	NA	NA		122	9.14		NA	NA	
>3 ANC visits	NA	NA		80	8.88		NA	NA	
Length of stay before delivery			NA			0.149			NA
0-2 days	NA	NA		170	9.07		NA	NA	
≥2 days	NA	NA		33	9.03		NA	NA	
Length of stay after delivery			NA			0.290			NA
0-1 day	NA	NA		120	8.99		NA	NA	
≥2 days	NA	NA		83	9.29		NA	NA	
Mode of transportation			0.043			0.798			0.177
Walking	232	8.94		66	8.98		154	9.01	
Motorized	156	9.17		137	9.14		76	8.78	
Wait time			0.613			0.143			0.902
≤1 hour	298	9.05		181	9.08		179	8.93	
>1 hour	90	8.98		22	8.73		51	8.96	
Self introduction by provider			0.003			0.176			<0.001
Not done	231	8.90		142	8.98		129	8.70	
Done	157	9.23		61	9.20		101	9.24	
Explanation of exam procedures (ANC, n = 380; Delivery, n = 121; PNC, n = 168)			0.003			0.991			0.029
Not explained	95	8.74		13	9.09		55	8.55	
Explained	285	9.13		108	9.08		113	9.01	
Explanation of medicine purpose & how to take (ANC, n = 366; Delivery, n = 167; PNC, n = 108)			0.009			0.273			0.014
Not explained	63	8.71		61	8.89		22	8.23	
Explained	303	9.12		106	9.06		86	9.05	
Explanation of blood specimen purpose (ANC, n = 267; Delivery, n = 85; PNC, n = 53)			0.012			0.098			0.001
Not explained	20	8.35		23	8.59		6	7.47	
Explained	247	9.04		62	9.10		47	8.60	
Consent seeking (ANC, n = 384; Delivery, n = 201; PNC, n = 183)			0.001			0.153			0.051
Not Sought	104	8.72		82	8.90		63	8.66	
Sought	280	9.15		119	9.12		120	9.03	
Encouragement to ask questions			<0.001			0.018			0.096
Not encouraged	143	8.76		123	8.90		107	8.80	
Encouraged	245	9.19		80	9.26		123	9.06	
Encouragement to have a guardian			0.596			0.007			0.096
Not encouraged	.	9.00		101	8.84		130	8.82	
Encouraged	208	9.06		102	9.24		100	9.09	
Confidentiality			0.001			0.016			0.105
Not Kept	24	8.33		17	8.45		30	9.27	
Kept	364	9.08		186	9.10		200	8.89	
Bp measurement ANC & PNC			0.027			NA			0.982
Not taken	147	8.88		NA	NA		133	8.94	
Taken	241	9.13		NA	NA		97	8.94	
Bp measurement before delivery			NA			0.927			NA
Not taken	NA	.		76	9.05		NA	NA	
Taken	NA	NA		127	9.04		NA	NA	
Bp measurement after delivery			NA			0.156			NA
Not taken	NA	NA		113	9.13		NA	NA	
Taken	NA	NA		87	8.92		NA	NA	
Baby Weight taken			NA			NA			0.248
Not taken	NA	NA		NA	NA		52	9.11	
Taken	NA	NA		NA	NA		178	8.89	

NA in all field of a cell means that this variable does not apply for the respective service

### Factors associated with perceived quality of conditions of the examination rooms

Bivariate analysis results of the associations between the perceived quality of room conditions and the independent variables are presented in Table 3. Literacy, previous still birth, number of ANC visits, and explanation of examination procedures during the clinical encounter were all statistically significantly associated with perceived quality of ANC services (i.e. p < 0.05). Specifically, literate women rated the quality of the examination room more highly. Women who had never had a still birth (as opposed to women who had had one already), women for whom the ANC visit was the first in their current pregnancy (as opposed to a follow-up visit), and women who had been explained the examination procedures (as opposed to not) also rated the quality of the examination room more highly. Perceived quality of delivery services was found to be significantly associated with being encouraged to ask questions during the clinical encounter (i.e. p < 0.05), in that women who had been encouraged rated the quality of the examination room more highly than women who had not been encouraged to ask questions. Perceived quality of PNC services was found to be significantly associated with explanation of the purpose of blood (i.e. p < 0.05). Specifically, women rated the quality of the examination room highly if the purpose of taking the blood specimen was explained, rather than not.

**Table 3:** Bivariate associations between the perceived quality of room conditions and the explanatory variables

Variable	ANC (N = 388)			Delivery (N = 203)			PNC (N = 230)		
	N	Mean	P-value	N	Mean	P-value	N	Mean	P-value
Age			0.700			0.812			0.503
<20	91	9.29		41	9.43		47	9.29	
20-29	205	9.36		118	9.31		132	9.13	
≥30	92	9.39		44	9.35		51	9.06	
Marital status			0.697			0.366			0.184
Unmarried	9	9.46		14	9.57		13	9.52	
Married	379	9.35		189	9.33		217	9.13	
Religion			0.448			0.449			0.779
Non-Christian	55	9.27		25	9.21		48	9.18	
Christian	333	9.36		178	9.36		182	9.14	
Literacy			0.032			0.250			0.392
Illiterate	131	9.22		69	9.23		89	9.07	
Literate	257	9.42		134	9.40		141	9.19	
Wealth			0.474			0.291			0.457
1 (poorest)	98	9.33		51	9.43		58	9.02	
2	97	9.34		54	9.13		57	9.06	
3	96	9.46		48	9.44		58	9.27	
4 (wealthiest)	97	9.28		50	9.40		57	9.24	
Parity			0.619			0.417			0.360
1	121	9.31		66	9.40		76	9.29	
2 to 3	135	9.33		74	9.22		86	9.08	
>3	132	9.41		63	9.42		68	9.08	
Number of children			0.538			0.376			0.234
No child	137	9.30		NA	NA		NA	NA	
1-3 children	189	9.36		149	9.31		178	9.19	
>3 children	62	9.44		54	9.44		52	9.00	
Miscarriage			0.905			0.696			0.330
No miscarriage	333	9.34		180	9.33		198	9.17	
Had miscarriage	55	9.36		23	9.42		32	8.98	
Still birth			<0.001			0.811			0.768
No stillbirth	371	9.38		197	9.34		221	9.15	
Had stillbirth	17	8.61		6	9.44		9	9.05	
Premature birth			0.460			0.594			0.984
No premature birth	365	9.34		191	9.33		215	9.15	
Had premature birth	23	9.49		12	9.49		15	9.15	
Number of current ANC visits			0.006			NA			NA
First visit	181	9.48		NA	NA		NA	NA	
>1 visit	207	9.24		NA	NA		NA	NA	
Method of delivery			NA			0.705			NA
Vaginal	NA	NA		190	9.35		NA	NA	
C-section	NA	NA		7	9.04		NA	NA	
Vacuum/forceps	NA	NA		4	9.41		NA	NA	
Number of ANC visits, delivery			NA			0.087			NA
0-3 ANC visits	NA	NA		122	9.44		NA	NA	
>3 ANC visits	NA	NA		80	9.20		NA	NA	
Length of stay before delivery			NA			0.491			NA
0-2 days	NA	NA		170	9.32		NA	NA	
≥2 days	NA	NA		33	9.35		NA	NA	
Length of stay after delivery			NA			0.129			NA
0-1 day	NA	NA		120	9.32		NA	NA	
≥2 days	NA	NA		83	9.45		NA	NA	
Mode of transportation			0.324			0.842			0.310
Walking	232	9.32		66	9.26		154	9.20	
Motorized	156	9.40		137	9.47		76	9.05	
Wait time			0.688			0.989			0.162
≤1 hour	298	9.34		181	9.34		179	9.20	
>1 hour	90	9.38		22	9.34		51	8.97	
Self introduction by provider			0.599			0.418			0.322
Not done	231	9.33		142	9.31		129	9.09	
Done	157	9.38		61	9.43		101	9.22	
Explanation of exam procedures (ANC, n = 380; Delivery, n = 121; PNC, n = 168)			0.033			0.275			0.487
Not explained	95	9.19		13	9.61		55	8.95	
Explained	285	9.40		108	9.29		113	9.08	
Explanation of medicine purpose & how to take (ANC, n = 366; Delivery, n = 167; PNC, n = 108)			0.809			0.383			0.343
Not explained	63	9.32		61	9.32		22	8.86	
Explained	303	9.36		106	9.29		86	9.22	
Explanation of blood specimen purpose (ANC, n = 267; Delivery, n = 85; PNC, n = 53)			0.066			0.347			0.004
Not explained	20	8.93		23	9.28		6	8.38	
Explained	247	9.36		62	9.49		47	8.80	
Consent seeking (ANC, n = 384; Delivery, n = 201; PNC, n = 183)			0.586			0.925			0.799
Not Sought	104	9.32		82	9.33		63	9.05	
Sought	280	9.37		119	9.34		120	9.10	
Encouragement to ask questions			0.061			0.012			0.398
Not encouraged	143	9.24		123	9.21		107	9.09	
Encouraged	245	9.41		80	9.55		123	9.20	
Encouragement to have a guardian			0.584			0.433			0.673
Not encouraged	180	9.33		101	9.29		130	9.12	
Encouraged	208	9.37		102	9.40		100	9.18	
Confidentiality			0.889			0.075			0.277
Not Kept	24	9.38		17	8.94		30	9.34	
Kept	364	9.39		186	9.38		200	9.12	
Bp measurement ANC & PNC			0.999			NA			0.725
Not taken	147	9.41		NA	NA		133	9.13	
Taken	241	9.37		NA	NA		97	9.18	
Bp measurement before delivery			NA			0.671			NA
Not taken	NA	NA		76	9.31		NA	NA	
Taken	NA	NA		127	9.37		NA	NA	
Bp measurement after delivery			NA			0.883			NA
Not taken	NA	NA		116	9.36		NA	NA	
Taken	NA	NA		87	9.33		NA	NA	
Baby Weight taken			NA			NA			0.872
Not taken	NA	NA		NA	NA		52	9.17	
Taken	NA	NA		NA	NA		178	9.14	

NA in all field of a cell means that this variable does not apply for the respective service

### Factors associated with perceived quality of nursing care services

Bivariate analysis results of the associations between the perceived quality of nursing care services and the independent variables are presented in Table 4. Literacy, consent seeking, explanation of medication, consent seeking and encouragement to ask questions were all statistically significantly associated with perceived quality of ANC services (i.e. p < 0.05). Specifically, literate women as well as women for whom the provider had done the above rated the quality of nursing care more highly than illiterate women and women who had not benefitted from the respective actions by the provider. Perceived quality of delivery services was found to be significantly associated with confidentiality being protected and blood pressure check after delivery (i.e. p < 0.05), in that women who had received the respective treatment by the provider rated the quality of nursing care more highly than those who had not. Perceived quality of PNC services was found to be significantly associated with wealth, religion, explanation of examination procedures, explanation of medication, explanation of the purpose of the blood specimen and consent seeking during the clinical encounter (i.e. p < 0.05). Specifically, non-Christian as well as wealthier women rated the quality of services more highly than Christians and poorer women. Further, women rated the quality of services more highly if the health worker explained procedures, purpose of medication and blood specimen and, sought consent as opposed to not having done so, respectively.

**Table 4:** Bivariate associations between the perceived quality of nursing care services and the explanatory variables

Variable	ANC (N = 388)			Delivery (N = 203)			PNC (N = 230)		
	N	Mean	P-value	N	Mean	P-value	N	Mean	P-value
Age			0.916			0.529			0.934
<20	91	9.05		41	9.27		47	8.73	
20-29	205	9.05		118	9.04		132	8.76	
≥30	92	8.99		44	9.13		51	8.83	
Marital status			0.550			0.991			0.609
Unmarried	9	9.22		14	9.12		13	8.95	
Married	379	9.03		189	9.10		217	8.76	
Religion			0.435			0.530			0.026
Non-Christian	55	9.13		25	8.97		48	9.14	
Christian	333	9.02		178	9.12		182	8.67	
Literacy			0.011			0.115			0.324
Illiterate	131	8.86		69	8.93		89	8.88	
Literate	257	9.12		134	9.20		141	8.70	
Wealth			0.194			0.624			0.046
1 (poorest)	98	9.01		51	9.05		58	8.40	
2	97	8.95		54	9.10		57	8.76	
3	96	9.21		48	9.28		58	8.89	
4 (wealthiest)	97	8.97		50	9.00		57	9.04	
Parity			0.734			0.508			0.622
1	121	8.98		66	9.24		76	8.68	
2 to 3	135	9.07		74	9.03		86	8.76	
>3	132	9.05		63	9.05		68	8.89	
Number of children			0.851			0.639			0.987
No child	137	9.00		NA	NA		NA	NA	
1-3 children	189	9.05		149	9.13		178	8.77	
>3 children	62	9.06		54	9.04		52	8.77	
Miscarriage			0.175			0.761			0.730
No miscarriage	333	9.06		180	9.11		198	8.78	
Had miscarriage	55	8.87		23	9.03		32	8.70	
Still birth			0.132			0.965			0.848
No still birth	371	9.05		197	9.10		221	8.77	
Had stillbirth	17	8.69		6	9.08		9	8.85	
Premature birth			0.587			0.495			0.358
No premature birth	365	9.23		191	9.09		215	8.75	
Had premature birth	23	9.32		12	9.32		15	9.07	
Number of current ANC visit			0.085			NA			NA
First visit	181	9.31		NA	NA		NA	NA	
>1 visit	207	9.17		NA	NA		NA	NA	
Method of delivery			NA			0.126			NA
Vaginal	NA	NA		190	9.35		NA	NA	
C-section	NA	NA		7	9.04		NA	NA	
Vacuum/forceps	NA	NA		4	9.41		NA	NA	
Number of ANC visits, delivery			NA			0.114			NA
0-3 ANC visits	NA	NA		122	9.44		NA	NA	
>3 ANC visits	NA	NA		80	9.20		NA	NA	
Length of stay before delivery			NA			0.602			NA
0-2 days	NA	NA		170	9.09		NA	NA	
≥2 days	NA	NA		33	9.20		NA	NA	
Length of stay after delivery			NA			0.123			NA
0-1 day	NA	NA		120	9.00		NA	NA	
≥2 days	NA	NA		83	9.25		NA	NA	
Mode of transportation			0.875			0.411			0.063
Walking	232	9.21		66	9.20		154	8.88	
Motorized	156	9.26		137	9.06		76	8.55	
Wait time			0.154			0.178			0.493
≤1 hour	298	9.27		181	9.14		179	8.74	
>1 hour	90	9.12		22	8.79		51	8.88	
Self introduction by provider			0.110			0.688			0.194
Not done	231	9.17		142	9.13		129	8.67	
Done	157	9.33		61	9.05		101	8.90	
Explanation of exam procedures (ANC, n = 380; Delivery, n = 121; PNC, n = 168)			0.129			0.874			0.001
Not explained	95	8.90		13	9.05		55	8.12	
Explained	285	9.07		108	9.11		113	8.83	
Explanation of medicine purpose & how to take (ANC, n = 366; Delivery, n = 167; PNC, n = 108)			0.036			0.169			0.002
Not explained	63	8.92		61	9.11		22	7.88	
Explained	303	9.09		106	8.99		86	8.81	
Explanation of blood specimen purpose (ANC, n = 267; Delivery, n = 85; PNC, n = 53)			0.212			0.629			<0.001
Not explained	20	8.68		23	8.90		6	7.39	
Explained	247	9.04		62	9.10		47	8.23	
Consent seeking (ANC, n = 384; Delivery, n = 201; PNC, n = 183)			0.008			0.977			0.035
Not Sought	104	8.83		82	9.09		63	8.40	
Sought	280	9.12		119	9.10		120	8.81	
Encouragement to ask questions			0.002			0.520			0.798
Not encouraged	143	8.84		123	9.06		107	8.75	
Encouraged	245	9.15		80	9.17		123	8.79	
Encouragement to have a guardian			0.413			0.068			0.581
Not encouraged	180	8.99		101	8.96		130	8.73	
Encouraged	208	9.07		102	9.25		100	8.82	
Confidentiality			0.059			<0.001			0.177
Not Kept	24	8.68		17	8.15		30	9.07	
Kept	364	9.06		186	9.19		200	8.73	
Bp measurement ANC & PNC			0.290			NA			0.239
Not taken	147	8.97		NA	NA		133	8.86	
Taken	241	9.07		NA	NA		97	8.65	
Bp measurement before delivery			NA			0.773			NA
Not taken	NA	NA		76	9.13		NA	NA	
Taken	NA	NA		127	9.09		NA	NA	
Bp measurement after delivery			NA			0.037			NA
Not taken	NA	NA		116	9.25		NA	NA	
Taken	NA	NA		87	8.91		NA	NA	
Baby Weight taken			NA			NA			0.075
Not taken	NA	NA		NA	NA		52	8.49	
Taken	NA	NA		NA	NA		178	8.85	

NA in all field of a cell means that this variable does not apply for the respective service

## Discussion

Overall, our study reveals that quality of interpersonal relations, room conditions, and general nursing care of all three services (ANC, delivery and PNC) were perceived to be good by the participants. In addition, the present study provides information for a better understanding of the factors that may be associated with perceived quality of maternal health care services.

Some socio-demographic factors were found to be more strongly associated with the perceived quality of interpersonal relations for ANC services than for delivery or PNC services. Literacy level served as an important determining factor insofar as those who were literate (in particular those with formal education) tended to rate the ANC service more highly than the illiterate. This finding is consistent with Lino et al. (2011) who suggested that women with a high level of education may have positive perceptions about the quality of ANC because they can judge and appreciate the benefits of ANC better [32]. Mode of transportation used to travel to the facility was also an important determinant, such that women that had used motorized transportation were more likely to rate the service highly than those who had walked. Distance to health facilities has been found to be one of the factors that impede accessibility in most developing countries [43]. Long travel times due to far distances and the resulting high effort to accessing health services may have contributed to a low rating of the service. In line with the study of Oladapo and Osiberu [16], our study did not find any association of the perceived quality of ANC care with other socio-demographic factors such as age, marital status and income.

Most service delivery factors were found to be strongly associated with perceived quality of interpersonal relations. Women tended to rate the ANC and PNC care highly if the health worker introduced himself/herself before attending to them. This seemed to be an issue of concern for ANC and PNC, but not for delivery, possibly because women usually come to the facility when they are already in labour and self-introduction may matter less to them than being assisted promptly. Still, according to the 2006 WHO guidelines on Pregnancy, Childbirth, Postpartum and Newborn Care, communication (which includes self-introduction) is an important aspect of quality [1]. Several other studies have supported this [20–22, 25, 26]. Moreover, the 2006 WHO guidelines and a prior study insist that patients have the right to know why certain examinations and medication are administered to them [1, 25]. In our study, we found that if explanations on examination procedures, purpose of medication and blood specimens were given during the consultation process, the more highly quality was rated on ANC and PNC services. This could be because the explanations made the women understand the importance of the basic procedures and their role in preventing a range of pregnancy complications and reducing maternal mortality [43]. Furthermore, our study revealed that being offered to have a guardian by one’s side during delivery increases the rating of quality of care. This finding supports the results of a prior study conducted in Malawi by Banda and colleagues which found that companionship or having a guardian during child-birth is important mainly for psychological and physical support to the labouring woman and for providing assistance to healthcare providers [44].

With regard to examination room conditions, women for whom the ANC visit was the first in their current pregnancy (as opposed to a follow-up visit), were more likely to rate highly the quality of care. A possible explanation could be that, apart from providers obtaining a large amount of information and conducting tests on the first visit, women are also supplied with their first dosage of ant-malarial drugs, Insecticides Treated Nets (ITN) and other supplies that may be available for pregnant women. Consequently, positive perceptions may have developed towards the rooms’ hygiene, comfort, and the availability of supplies [23, 25].

Regarding the nursing care services, women that received an explanation of the purpose of the medication given and were encouraged to ask questions during the ANC consultations were more likely to rate the quality of care highly. This might be explained by the women’s wish to understand what is happening to them and their unborn babies. The question and answer process promotes learning of information important for positive health outcomes [45]. Furthermore, our study revealed consent seeking as an important factor that may influence perceived quality of care. The more consent was sought from the women; the more highly they rated nursing care services on ANC and PNC services. Further, the more the purpose of examination, medication and blood specimen was explained the more highly they rated nursing care services on PNC services. In addition, privacy and confidentiality issues served as important determining factors for perceived quality of the nursing care services in delivery services. This finding is consistent with Jallow et al. (2012) who found inadequate privacy to be associated with women’s poor perceptions of ANC services in Gambia [21].

The client perception score based on experiential scaling appears to be a psychometrically reliable and valid instrument for use. However, for the general use of this multi-dimensional instrument, content validity of single experience statements would need to be considered. For the purpose of our study, we mainly relied on existing literature and a similar tool used in another African setting in order to ensure the content to be sufficiently valid. In an ideal setting, however, preceding qualitative assessment of relevant experiential dimensions in combination with expert input on more ideal conceptual approaches linking client experiences with perception dimensions could allow more defined results [46]. Further, the use of either a sequential item approach or a Comprehensive Exploratory Factor Analysis (CEFA) may be a useful strategy to improve the validity of the tool [47].

### Limitations of the study

The study used convenience sampling, including only participants who presented themselves at the facility during the three days of the visit of our study team; women attending on other days may have different experiences and perceptions about the quality of care services. For example, women not attending Friday clinics may be Muslims; and that the experiences on the day may be dependent on the health worker available on the day; and the conduct of a particular health worker today may not be their usual conduct. As such, the results may not generalize to the entire population.

Furthermore, the overall results demonstrate that the quality of care was perceived to be good, considering the high level of the overall perceived quality of care mean scores of 9 as compared to a possible score of 10. These findings should be interpreted with caution, not jumping to the immediate conclusion that quality of maternal care service is impeccable in Malawi. Several factors that might have influenced the ratings ought to be considered. First, participants may have over-rated the quality of the services because they were interviewed at the facility, fearing to be overheard by healthcare providers or other clients, but also out of ignorance of what constitutes the ideal. As suggested by Kumbani et al. (2012), women in Malawi may not be critical of the care they receive because they are not aware of the quality of care to expect and because of their lack of awareness on prescribed standards of care [23]. In addition, often times participants will appreciate the services for politeness’ sake or for fear that the service may be withdrawn from them, and respond favourably to questions [21]. Moreover, in situations where people do not know their health-related rights, they are likely to accept whatever service is given to them [48]. This is a significant reason why human rights-based approaches to addressing maternal mortality are advocated, in order to empower and support women in claiming their right to maternal health [49]. Second, the use of a quantitative tool to elicit perceptions might have challenged the women’s ability to explicitly express the complexity of their judgement on the quality of care received, forcing them to respond only to narrowly formulated statements. It follows that our preliminary quantitative work needs to be complemented by a further qualitative study to look into the complexity that quantitative data cannot unravel.

## Conclusion

Our study suggests that women’s socio-demographic factors and their experiences at the health facility have a great influence on their perceptions about quality of care. Assuming that perceptions are important determinants for future utilization, one solution to non-utilization of maternal and newborn care may lie in the improvement of how clients perceive quality of care. The factors that have been identified in this study to be influencing women’s perceived quality of care are important for service delivery improvement and utilization. The information generated by this study will be useful in planning and improving the effectiveness and quality of care by the Malawi government. It is essential that government policies direct more emphasis to stay on track in raising the standards of quality; strengthen maternal and newborn care programs; and encourage attendance of ANC, skilled birth, newborn care and PNC services in order to reduce preventable mortality and improve health for women and their babies.

## Additional files

Additional file 1:
**ANC Exit Interview Questionnaire.**
Additional file 2:
**Delivery Exit Interview Questionnaire.**
Additional file 3:
**PNC Exit Interview Questionnaire.**

## Abbreviations

ANC: Antenatal Clinic
BP: Blood Pressure
CD: Comparative Data
CFA: Confirmatory Factor Analysis
LMICs: Low and Middle Income Countries
PNC: Postnatal Clinic
SD: Standard Deviation
SRMR: Standardized Root Mean Square Residual
RMSEA: Root Mean Square Error of Approximation.
